# Transcriptome analysis reveals Vernalization is independent of cold acclimation in *Arabidopsis*

**DOI:** 10.1186/s12864-021-07763-3

**Published:** 2021-06-21

**Authors:** Fei Li, Qian Hu, Fadi Chen, Jia Fu Jiang

**Affiliations:** State Key Laboratory of Crop Genetics and Germplasm Enhancement, Key Laboratory of Landscaping, Ministry of Agriculture and Rural Affairs, Key Laboratory of Biology of Ornamental Plants in East China, National Forestry and Grassland Administration, College of Horticulture, Nanjing Agricultural University, Nanjing, 210095 China

**Keywords:** *Arabidopsis*, Transcriptome profiling, Cold stress, Vernalization, *FLC*

## Abstract

**Background:**

Through vernalization, plants achieve flowering competence by sensing prolonged cold exposure (constant exposure approximately 2-5 °C). During this process, plants initiate defense responses to endure cold conditions. Here, we conducted transcriptome analysis of *Arabidopsis* plants subjected to prolonged cold exposure (6 weeks) to explore the physiological dynamics of vernalization and uncover the relationship between vernalization and cold stress.

**Results:**

Time-lag initiation of the two pathways and weighted gene co-expression network analysis (WGCNA) revealed that vernalization is independent of cold acclimation. Moreover, WGCNA revealed three major networks involving ethylene and jasmonic acid response, cold acclimation, and chromatin modification in response to prolonged cold exposure. Finally, throughout vernalization, the cold stress response is regulated via an alternative splicing-mediated mechanism.

**Conclusion:**

These findings illustrate a comprehensive picture of cold stress- and vernalization-mediated global changes in *Arabidopsis*.

**Supplementary Information:**

The online version contains supplementary material available at 10.1186/s12864-021-07763-3.

## Introduction

Plants are sessile organisms that passively sense environmental signals, such as temperature. Vernalization is a process through which plants achieve flowering following prolonged cold exposure. Typically, winter annual accessions of *Arabidopsis* require several weeks of cold exposure before flowering, whereas its rapid-flowering accessions do not require vernalization. The difference between the two accessions is determined by the expression of the dominant allele of FRIGIDA (*FRI*) [1, 2]. *FRI* encodes a scaffold protein that functions as an activator of FLOWERING LOCUS C (*FLC*). *FLC* encodes a MADS-BOX transcription factor that functions as a suppressor of the floral integrators FT and SUPPRESSOR OF OVEREXPRESSION OF CO 1 (SOC1) [3].

Before vernalization, *FLC* chromatin is in an active transcription state during vegetative growth. FRI forms a complex with FRI-LIKE 1 (FRL1), FRI ESSENTIAL 1 (FES1), SUPPRESSOR OF FRI 4 (SUF4), and FLC EXPRESSOR (FLX) to recruit other transcription factors and chromatin modifiers, ultimately activating *FLC* [4]. Upon cold exposure, *FLC* suppression is initiated via upregulation of the noncoding *FLC* antisense transcript *COORAIR* [5]. Subsequent suppression is realized by dynamic replacement of active markers via trimethylation of lysine 27 of histone H3 (H3K27me3) by Polycomb repressive complex 2 (PRC2) [6]. PRC2 is conserved in both animals and plants Homologs of the *Drosophila* H3K27 methyltransferase E(z), namely CURLY LEAF (CLF) and SWINGER (SWN), together with VERNALIZATION 2 (VRN2), FERTILIZATION INDEPENDENT ENDOSPERM (FIE), and SUPPRESSOR OF IRA 1 (MSI1) constitute the core components of PRC2 [7, 8]. VIN3, encoding a chromatin-remodeling plant homeodomain (PHD) finger protein, forms a heterodimer with the paralog VIN3-like 1 (VIL1)/VERNALIZATION 5 (VRN5) and joins the PRC2 core to serve as the cold-specific PHD (VIN3)-PRC2. Moreover, Polycomb partners VAL1 and VAL2 serve as epigenome readers that recognize the *cis*-regulatory element at the FLC locus to recruit histone deacetylases HDA9 and PRC2. The former catalyzes H3K27ac deacetylation to H3K27, and the latter catalyzes H3K27 trimethylation to H3K27me3, synergistically inhibiting FLC expression to promote flowering [9]. As the temperature increases (22 °C), LIKE HETEROCHROMATIN PROTEIN 1 (LHP1), and VRN2 recognize H3K27me3, thus stably maintaining *FLC* suppression. Multiple genes alter the chromatin structure of the FLC locus to inhibit *FLC* expression [10, 11].

The entire process of vernalization requires at least 1 month of cold exposure for enabling plants to saturate the vernalization response. During this period, plants must also initiate cold acclimation to endure harsh environments. Cold acclimation is a rapid adaptive response that enables plants to acquire freezing tolerance. In most cases, 1-2 d of exposure to low but non-freezing temperatures is sufficient for plants to acquire cold acclimation [12]. *A. thaliana* achieves maximum freezing tolerance after 7 d of exposure to temperatures as low as 2 °C [13, 14]. When cold-acclimated plants are treated with non-acclimation-inducing temperatures, they lose their freezing tolerance. This is known as deacclimation [15]. The required duration for deacclimation is much shorter. For instance, 24 h is sufficient to deacclimatize *Arabidopsis* [15]. There are two basic types of cold-response pathways that enable plants to achieve cold acclimation, namely CBF-dependent and CBF-independent pathways. ICE1-CBF-COR is the core model of the CBF-dependent pathway. Inducer of CBF expression 1 (ICE1) is an MYC-like basic helix–loop–helix transcription factor that can bind to the MYC *cis*-acting elements in the CBF promoter [16, 17]. HIGH EXPRESSION OF OSMOTICALLY RESPONSIVE GENE1 (HOS1), SAP AND MIZ1 DOMAIN-CONTAINING LIGASE 1 (SIZ1), and OPEN STOMATA 1 (OST1) regulate ICE1 through ubiquitination, sumoylation, and phosphorylation, respectively, thus participating in the CBF-dependent pathway [18–20]. The *COR* genes comprise four gene families, namely low temperature-inducible (LTI), cold-inducible (KIN), responsive to desiccation (RD), and early dehydration-inducible (ERD) genes [21]. Of note, 10% of all *COR* genes are regulated by CBF1, CBF2, and CBF3 (or DREB1b, DREB1c, and DREB1a, respectively [22, 23]. At low but non-freezing temperatures, ICE1 directly binds to the *CBF* promoter to activate its expression, further enhancing *COR* expression to enhance cold tolerance [24–26].

Phytohormones are essential in the regulation of freezing tolerance. JA positively regulates the ICE–CBF pathway to enhance freezing tolerance in Arabidopsis [27]. Blocking JA biosynthesis and signaling produces hypersensitivity to freezing tolerance [27]. The ethylene signaling pathway negatively regulates freezing tolerance. EIN3 suppresses the expression of CBFs and Type-A ARR genes by directly binding to their promoters [24, 28].

The relationship between cold stress response and vernalization in plants remains controversial. In 2004, Sung and Amasino [12] reported that one distinction between cold acclimation and vernalization is the time lag between the two pathways (approximately 10 d) with *Col*-FRI seedlings. As such, vernalization occurs approximately 10 d later than cold acclimation, based on the induction time of *VIN3* [12, 29]. As the first gene of the vernalization pathway, *VIN3* can be detected within 1 day of cold treatment in the background of rapid-flowering ecotypes, whereas in *Col*-FRI, *VIN3* can only be induced by cold exposure longer than 2 weeks [29, 30]. In 2009, Seo et al. [31] reported that CBFs could induce *FLC* expression under intermittent cold (0–6 h), thereby delaying flowering. Vernalization could override this effect by inhibiting *FLC* expression, demonstrating a complicated relationship between these two pathways. HOS1 is a negative regulator of cold responses. The demonstration that HOS1 can regulate FLC expression through chromatin remodeling under cold temperatures providing new insights into the crosstalk between the two pathways [32]. However, Bond et al. [33] illustrated the independence of  cold acclimation and vernalization by showing that none of the key components of cold acclimation signaling, such as *ICE1* and *HOS1*, play a role in VIN3 induction. To this end, in the present study, we conducted transcriptome analysis to explore the relationship between vernalization and cold acclimation.

## Results

### Transcriptional dynamics of Vernalization in Arabidopsis

**Fig. 1:**
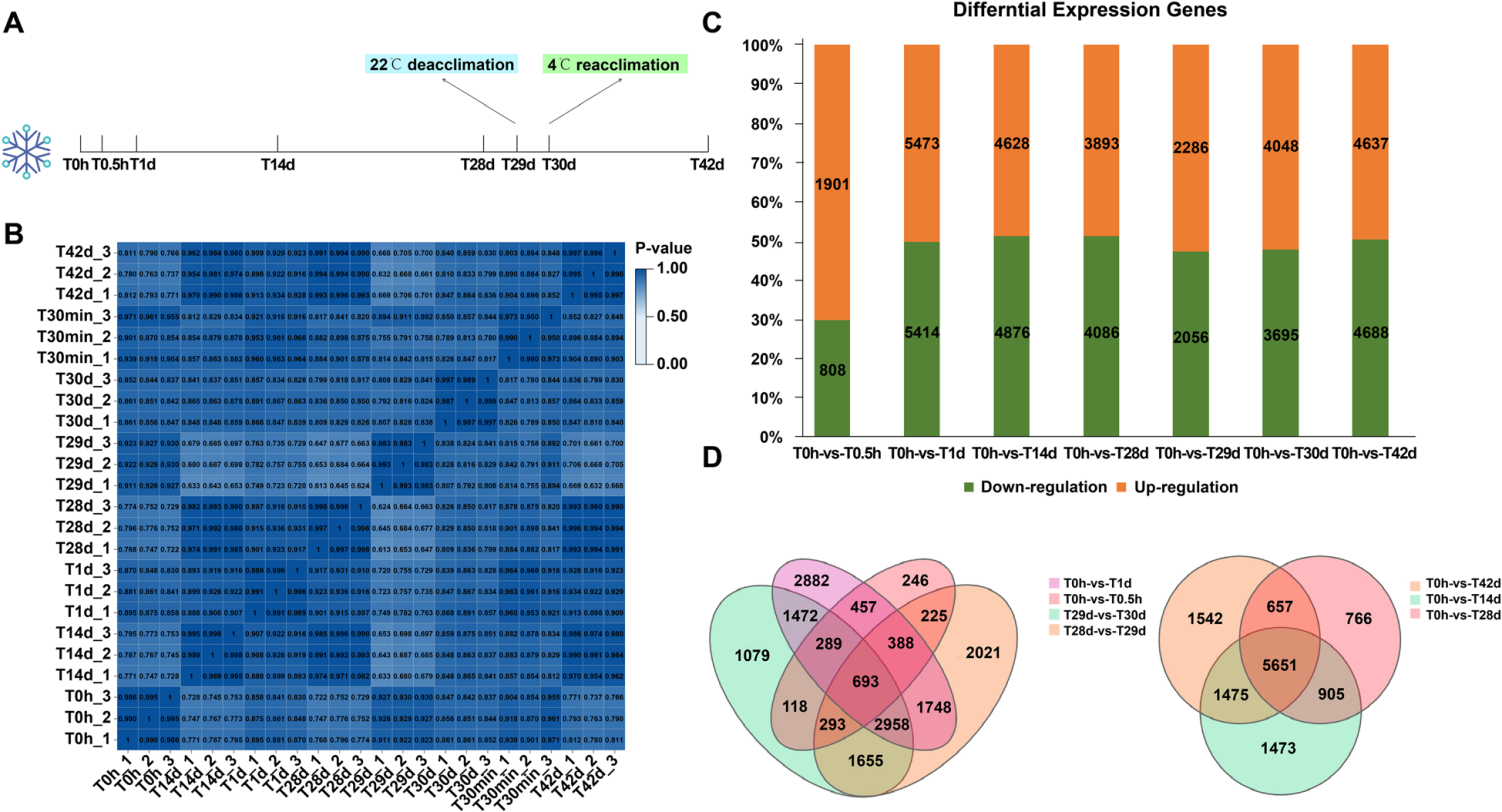
Experiment overview and effect of vernalization. **A ***Arabidopsis* FRI-Col (*Col-0* with a functional *FRI* allele) was used in this experiment. Seedlings were incubated in growth chambers at 22 °C under a 16-8 h day-night period for about 2 wk. (until they grew two true leaves) and then harvested on March 23 (T0h). **B** Heatmap depicting pairwise Pearson correlation of gene expression values of all samples. **C** Bar graph showing total number of differentially upregulated (orange) and downregulated (green) genes in T0hVS0.5 h, T0hVS1d, T0hVS14d, T0hVS28d, T0hVS29d, T0hVS30d, and T0hVS42d samples. **D** Venn diagram showing common and unique genes in T0hVST0.5 h, T0hVST1d, T29VST30d, T0hVST14d, T0hVST28d, and T0hVST42d samples

With the aim of profiling the whole picture of transcriptional dynamics during vernalization and cold stress response in *Arabidopsis*, we conducted transcriptome analysis of plants exposed to 42 d of cold required to saturate the vernalization response in *Col-FRI* [34]. We harvested whole plants of FRI-*Col* (Col-0 with a functional *FRI* allele) and set up eight sampling time points, four of which (0 d, 14 d, 28 d, and 42 d) were designed to explore the effects of vernalization and the remaining four (0.5 h, 1 d, 29 d, and 30 d) to explore the effects of cold stress. Of note, the 29 d samples were subjected to 1 d of deacclimation (22 °C) and the 30 d samples were subjected to 1 d of reacclimation (Fig. 1a). Here, we focused on the cold exposure phase of vernalization, and 1 d was sufficient to deacclimate but not to devernalize *Arabidopsis*. The BGISEQ-500 was used to detect differentially expressed genes (DEGs). The correlation heatmap showed that the T14d, T28d, T42d, T0h, and T29d transcriptomes were similar to one another (Fig. 1b). A total of 31,744 DEGs were identified. At the initiation stage of cold exposure, the expression of only 2709 genes was altered within 0.5 h, and the expression level in these samples was almost one-fourth of that in the 1 d samples (Fig. 1c). In addition, the highest number of DEGs was detected in the 1 d samples, indicating that 1 d was the most drastic response time. Genes in the 30 d samples exhibited no significant changes, even though they experienced 1 d of cold exposure after recovery, which may be explained by the gain of cold acclimation as the plants had already been exposed to cold for 28 d. The number of DEGs was similar in the 14, 28, and 42 d samples, suggesting that plants maintained a high level of response to prolonged cold exposure (Fig. 1c). Among the short-term cold exposure treatments, the T0hVST0.5 h, T0dVST1d, and T29dVST30d samples shared only 982 DEGs. Among long-term cold exposure treatments, the T0hVST14d, T0VS28d, and T0VS42d samples shared 5651 DEGs. These results indicate that short-term cold response is more flexible, whereas long-term cold response is more stable (Fig. 1d).

**Fig. 2:**
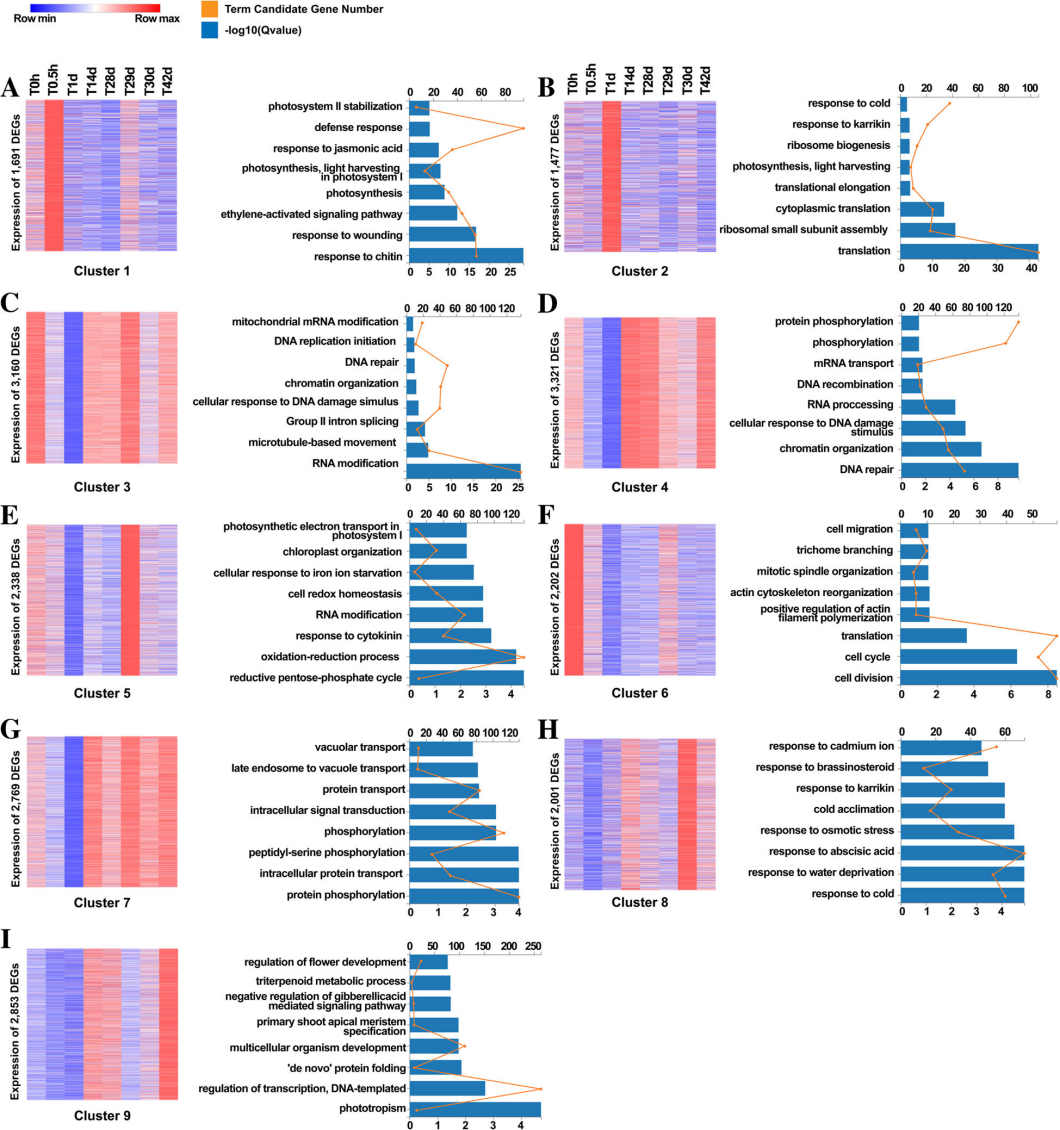
Time-course analysis of dynamic gene expression changes during vernalization. **A-I** From left to right: heat map showing expression patterns of cluster 1–9 genes and Gene Ontology (GO_P) terms of each cluster. Clustering was performed using Mfuzz [35]. Expression level of genes in clusters 1–9were normalized to Log_2_(FPKM+ 1); red and blue represent up- and downregulated genes, respectively

Time-course analysis was conducted by clustering all genes from different time points to investigate their expression dynamics (Fig. 2). Genes in clusters 1 and 2 showed a rapid response to cold within 0.5 h and 1 d, respectively (Fig. 2a, b). Gene Ontology (GO) analysis indicated that genes involved in response within 0.5 h were sensitive to stress and were enriched in wounding response, defense response, and ethylene-activated signaling pathways (Fig. 2a). Genes involved in response within 1 d were associated with ribosomal assembly and protein translation (Fig. 2b). Genes in clusters 3 and 4 showed similar expression patterns (Fig. 2c, d), exhibiting a rapid response to temperature change at 0.5 h and 29 d, respectively (Fig. 2c, d). GO analysis indicated that the upregulated genes in these two clusters were enriched in DNA repair and RNA modification, perhaps because such a lasting response can induce DNA damage and enhance replication (Fig. 2c, d). Genes in clusters 5 and 6 were downregulated during cold exposure and were sensitive to increases in temperature (Fig. 2e, f). GO analysis revealed that genes in cluster 5 were enriched in the reductive pentose phosphate cycle, redox processes, and cytokinin response, while those in cluster 6 were enriched in cell division and cell cycle. These results indicate that the cell cycle, energy consumption, and oxidative activity are effective at relatively high temperatures, but are suppressed at low temperatures (Fig. 2e, f). Genes in cluster 7 maintained high expression after 1 d of cold exposure (Fig. 2g) and were primarily enriched in phosphorylation-related processes, including the MKK and CIPK9 signaling pathways as well as intracellular protein transport (Fig. 2g). This result suggests that phosphorylation plays an important role in the response to long-term temperature changes. Genes in cluster 8 were enriched in cold response and cold acclimation (Fig. 2h); the expression of genes involved in cold response peaked at 14 d and stably dropped thereafter, whereas that of genes involved in cold acclimation peaked at 42 d. Genes in cluster 9 were involved in the regulation of flower development (Fig. 2i). In addition, genes in cluster 8 were sensitive to temperature change, but those in cluster 9 showed no such activity. Collectively, these expression patterns indicate the primary relationships and differences between short- and long-term responses to cold (Fig. 2h, i) (Table S1).

### Relationship between cold acclimation and Vernalization

**Fig. 3:**
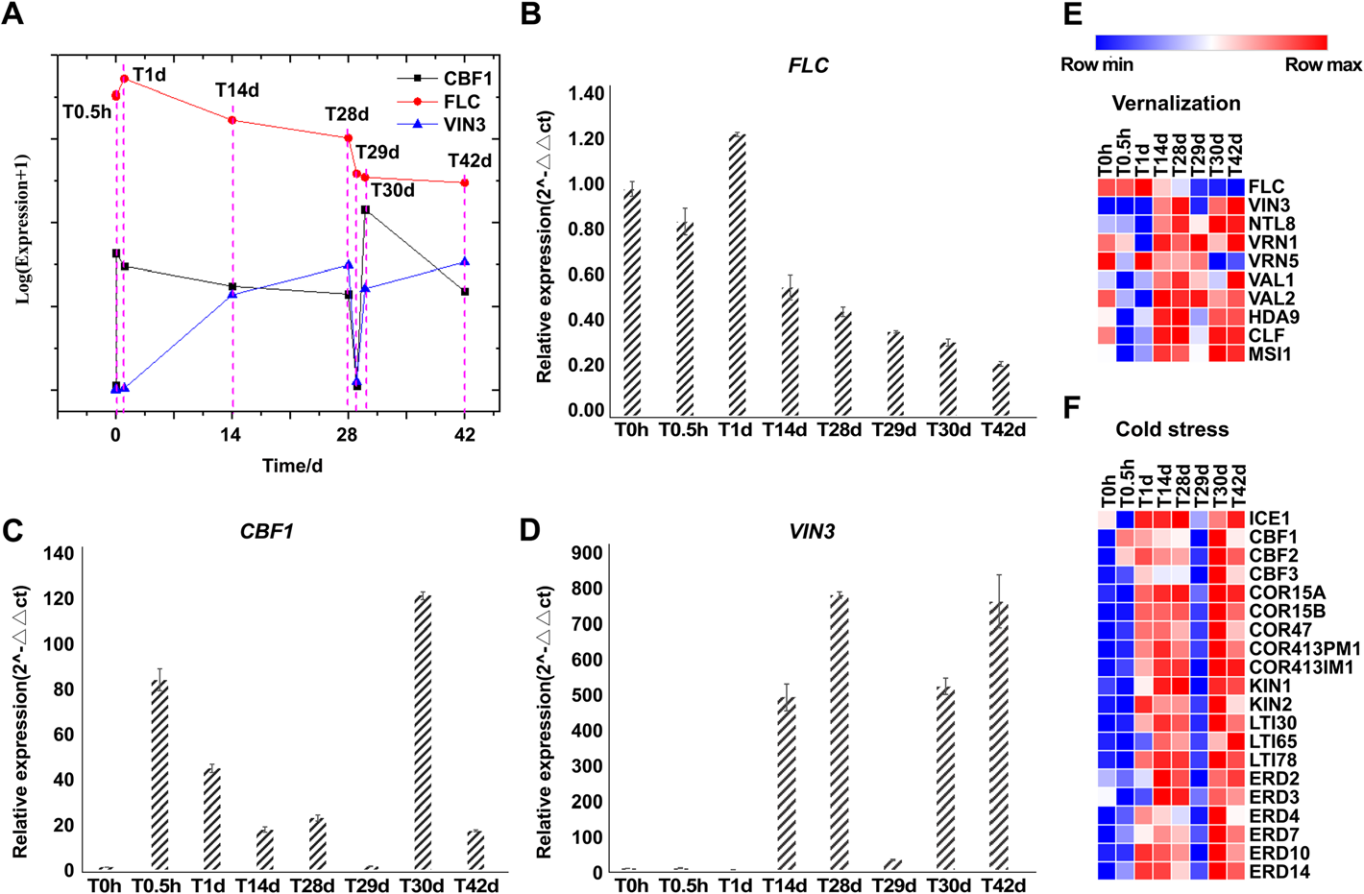
Different expression patterns of *FLC*, *VIN3*, and *CBF1* and heatmap of genes involved in the vernalization and cold acclimation pathways. **A** Line chart showing expression patterns of *FLC*, *VIN3*, and *CBF1* throughout vernalization [fragments per kilobase per million (FPKM)]. Data were normalized to Log_2_(FPKM+ 1). **B-D** RT-qPCR validation of *FLC*, *VIN3*, and *CBF1* at different time points. Values shown are means (*n* = 3). Error bars indicate SE (*n* = 3). **E-F** Heatmap showing expression patterns of genes involved in vernalization and cold stress response. Gene expression data were normalized to Log_2_(FPKM+ 1); red and blue represent up- and downregulated genes, respectively. Box-plots showing expression patterns of genes involved in vernalization and cold stress response. Gene expression data were normalized to Log_2_(FPKM+ 1)

Cold acclimation and vernalization were likely initiated with a time lag of approximately 10 d. To explore the overlaps and interactions between cold acclimation and vernalization, we focused on genes involved in the relevant pathways (Table S2). The expression of *CBF1*, which is the key gene involved in cold stress response, peaked within 0.5 h and remained stable thereafter (Fig. 3a). The expression of other genes known to be involved in the cold response also showed a typical upward trend. *ICE1*, *COR15A*, *COR15B*, *COR47*, *COR413PM1*, *COR413IM1*, *LTI30, LTI65, LTI78*, *ERD2*, *ERD3*, *ERD4*, *ERD7*, *ERD10*, *ERD14*, *KIN1*and *KIN2* were significantly upregulated within 1 d (Fig. 3f) (Table S2). Conversely, the expression of *FLC*, which is the key gene involved in vernalization, was initially suppressed at 14 d, along with the induction of *VIN3*, which is considered the first gene activated in the vernalization pathway (Fig. 3a). In addition, *NTL8*, which was recently shown to upregulate *VIN3* under long-term cold [36], showed similar expression to *VIN3*. The expression of the PRC2 genes showed a typical upward trend. *VAL1* expression showed an obvious upward trend during vernalization, while *VAL2* expression dropped to the normal level after a slight increase (Table S2). Notably, the expression of almost all genes in the vernalization pathway was altered to regulate *FLC* expression after 14 d (Fig. 3e). Box plots showed that the expression patterns of genes involved in the two pathways were distinct in terms of the time point of their change (1 d for cold acclimation and 14 d for vernalization) (Fig. 3g, h). We confirmed this observation using qPCR, and the patterns of *FLC*, *CBF1*, and *VIN3* expression were consistent between qPCR and RNA-seq (Fig. 3b, c, d).

HOS1 is a crosstalk gene between cold stress and vernalization, which regulates *FLC* expression under intermittent cold conditions and physically interacts with ICE1 to mediate its ubiquitination [18, 32]. The constant increase in *FLC* expression and decrease in *ICE1* expression within 1 d may be attributed to this function of HOS1. However, the upward trend of *FLC* expression and the downward trend of *ICE1* expression did not last long. After 1 d of cold exposure, the expression of these two genes showed converse trends, indicating that such interactive activity would not affect the timing of initiation of the two pathways. Therefore, consistent with the time-lag notion, rapid response genes are not involved in quantitative response, that is, cold acclimation and vernalization are independent from the perspective of overlapping regulatory genes.

#### Network analysis of cold stress and Vernalization based on weighted gene co-expression network analysis (WGCNA)

**Fig. 4:**
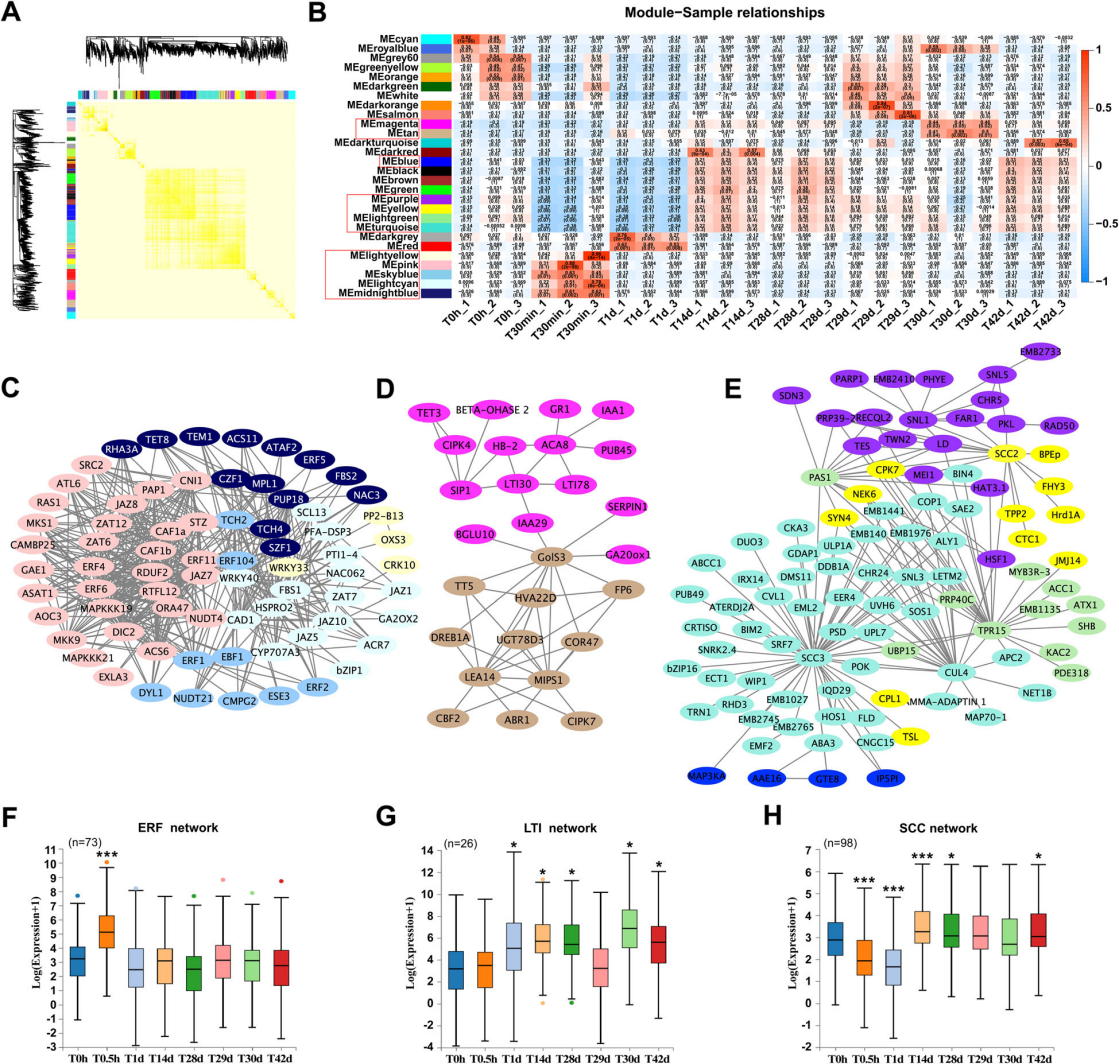
Weighted gene co-expression network analysis (WGCNA). **A** Heatmap depicting the topological overlap matrix among all genes in the analysis. Blocks of darker colors along the diagonal indicate 28 modules. **B** Heatmap showing module–sample correlation. **C** The correlation networks of major genes from the lightyellow, pink, skyblue, lightcyan, and midnightblue modules. **D** The correlation network of major genes from the magenta and tan modules. **E** The correlation network of major genes from the blue, purple, lightgreen, yellow, and turquoise modules. **F-G** Box-plots showing expression pattern of each network. The x-axis indicates time points (0 h, 0.5 h, 1 d, 14 d, 28 d, 29 d, 30 d, and 42 d), and gene expression data were normalized to Log_2_(FPKM+ 1). Asterisk is significantly different at P < 0.05 (Student’s t test)

Furthermore, WGCNA was performed to explore the relationship between cold stress and vernalization. The analysis yielded 28 modules (Fig. 4a and b; Table S3). Three core networks were identified as the key models of cold response, and genes with known functions were selected. We designated these models as the ERF, LTI, and SCC networks based on hub genes with high module membership (MM) values (Table S4). Genes with MM values over 0.98, are listed in Table 1. Genes in the ERF network belonged to cluster 1 and were involved in an instant cold response. Consistent with the GO_P analysis of cluster 1, the ERF network included ethylene and jasmonic acid (JA) response factors, with the hub genes *ERF11*, *ERF104*, *JAZ7*, and *WRKY40* (MM > 0.99) (Fig. 4c). ETHYLENE RESPONSIVE ELEMENT-BINDING FACTORs (ERFs) are the core ethylene response factors, and five ERFs, namely *ERF1*, *ERF2*, *ERF4*, *ERF5*, and *ERF6*, were also involved in this network. JASMONATE-ZIM-DOMAIN PROTEINs (JAZs) play pivotal roles in JA signaling, and five JAZs, namely *JAZ1*, *JAZ5*, *JAZ7*, *JAZ8*, and *JAZ10*, were involved in this network (Fig. 4c). Additionally, the ethylene biosynthesis genes *ACS6* and *ACS11* and the JA biosynthesis gene *AOC3* functioned at the relative outer circle of the ERF network, suggesting that ethylene and JA are the key hormones for short-term cold response. *ZAT6*, *ZAT7*, and *ZAT12* of the C2H2 ZINC FINGER TRANSCRIPTION FACTOR (ZFP) family were also part of the ERF network, and these genes were primarily involved in the stress response (Fig. 4c).

**Table 1:** Genes with module membership (MM) values over 0.98

Gene ID	Gene Symbol	Module	MM value	***P***-value
839,733	*ERF11*	pink	0.99	4.64e-24
836,281	*ERF104*	skyblue	0.99	2.01e-21
818,025	*JAZ7*	pink	0.99	5.48e-21
844,423	*WRKY40*	lightcyan	0.99	2.63e-20
837,071	*LEA14*	tan	0.99	6.57e-26
824,261	*LTI30*	magenta	0.98	5.73e-17
819,410	*SCC3*	turquoise	0.99	8.66e-25
831,407	*SCC2*	yellow	0.99	1.33e-22
818,750	*TPR15*	lightgreen	0.99	2.05e-23
821,265	*SNL1*	purple	0.99	6.73e-22
834,663	*CUL4*	turquoise	0.99	4.06e-21
839,666	*STZ*	pink	0.98	1.13e-19
839,846	*CAD1*	lightcyan	0.98	1.2e-19
838,410	*NUDT4*	pink	0.98	3.94e-19
842,428	*FBS1*	lightcyan	0.98	8.79e-19
5,008,138	*RTFL12*	pink	0.98	8.95e-19
836,074	*RDUF2*	pink	0.98	3.08e-18
823,551	*CAF1a*	pink	0.98	1.16e-18
832,285	*CAF1b*	pink	0.98	2.34e-17
818,429	*WRKY33*	lightyellow	0.98	1.36e-17
835,860	*TCH4*	midnightblue	0.98	5.32e-17
843,832	*ORA47*	pink	0.98	6.61e-18
835,815	*ACA8*	magenta	0.98	2.49e-18
818,568	*HOS1*	turquoise	0.98	2.83e-17
818,459	*EMB2765*	turquoise	0.98	3.83e-18
814,729	*SOS1*	turquoise	0.98	1.32e-17
844,068	*MEI1*	purple	0.98	5.51e-17

The LTI network involved typical cold acclimation genes from cluster 8. The expression levels of genes in T14d, T28d, T30d, and T42d samples were significantly different from those at T0h (Fig. 4g) (Table S4). The hub genes of this network included LOW TEMPERATURE INDUCED 30 (*LTI30*), LATE EMBRYOGENESIS ABUNDANT 14 (*LEA14*), and *A. thaliana* ALPHA CARBONIC ANHYDRASE 8 (*ACA8*) (MM > 0.98) (Fig. 4d). Notably, IAA1 and IAA29 were also part of this network, indicating that auxin is involved in cold acclimation.

The SCC network was the largest model detected in the present study. Genes in this network were first downregulated at T0.5h and T1d, and then upregulated at T14d, T28d, and T42d, indicating that this network is likely involved in response to long-term cold (Fig. 4h; Table S4). *SCC2*, *SCC3*, and *CUL4* (MM > 0.99) were the hub genes of the SCC network (Fig. 4e). CUL4 is one of the CULLIN (CUL) RING UBIQUITIN LIGASEs (CRLs), which are involved in substrate ubiquitination [37]. *SCC2* and *SCC3* are essential for maintaining centromere cohesion during anaphase I. Many genes from the SCC network are involved in chromatin modification. These included *HOS1*, the trimethylase *ATX1*, *LD*, and *FLD* from the autonomous flowering pathway, *SNL1* related to deacetylation, and others [32, 38–41] (Fig. 4e). These three networks represent three major parts of the cold response. The first is an instantaneous response, particularly at T0.5h, which is related to JA/ethylene signaling. The second is a rapid and lasting response that is sensitive to 28-30d temperature change, which is related to cold acclimation pathway. The third is a stable response featuring a consistent expression level at T14d, T28d, and T42d, which is related to chromatin modification (Fig. 4f, g, h). Notably, the *VIN*3 and *FLC* as core genes of vernalization were not involved in any module of WGCNA, indicating that the cold response is independent of vernalization.

#### Alternative splicing mediation during Vernalization

**Fig. 5:**
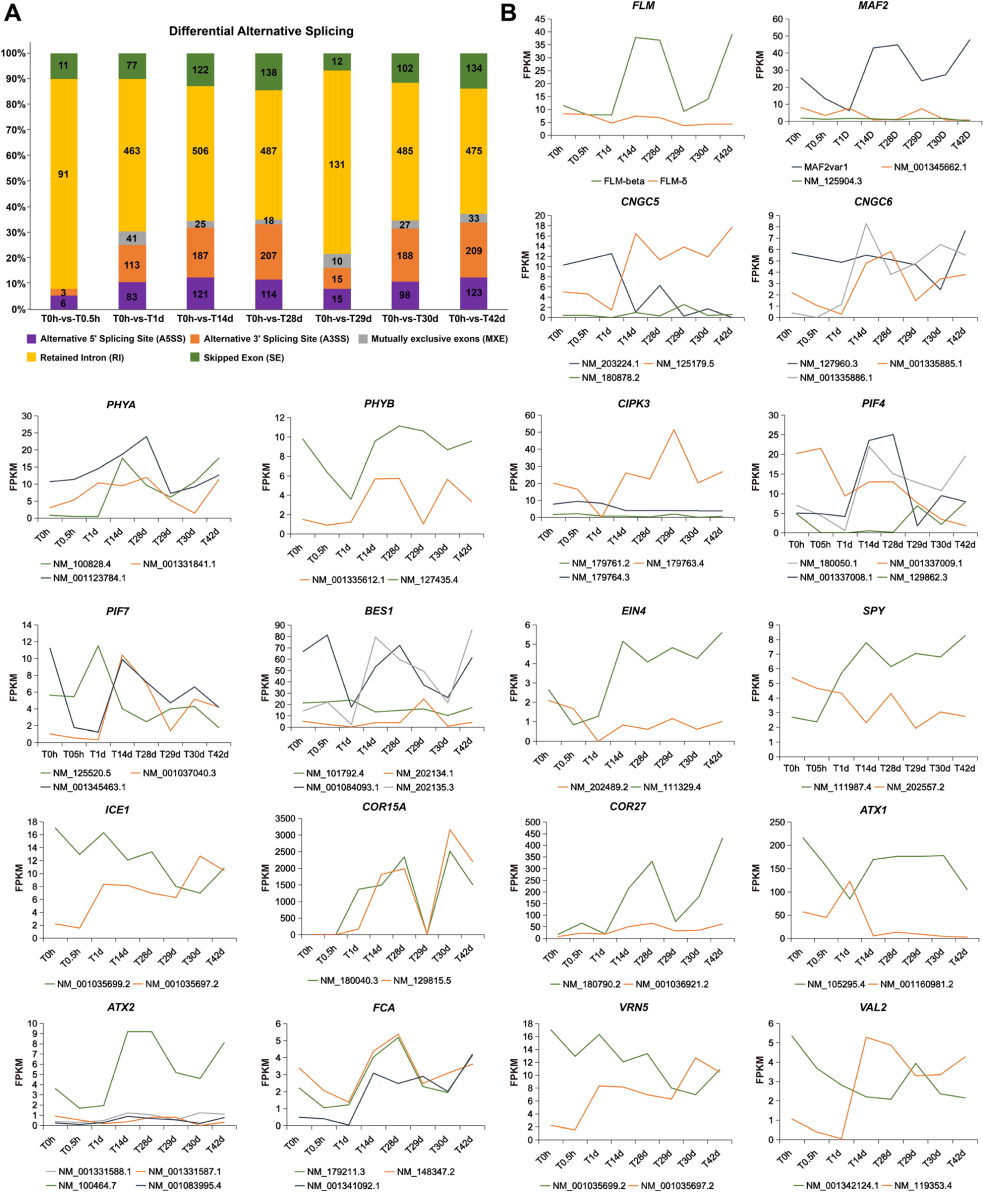
Alternative splicing of some differentially expressed genes (DEGs) during cold exposure. **A** Bar graph showing total number of differentially alternative splicing genes of five types of mechanisms (skipped exons, mutually exclusive exons, alternative 3′/5′ splicing sites, and retained introns) at 0.5 h, 1 d, 14 d, 28 d, 29 d, 30 d, and 42 d. **B** Line charts showing expression patterns of different transcripts of selected genes

Alternative splicing is a ubiquitous co-transcriptional RNA modification through which multiple transcripts can be generated from a single gene. Temperature is closely associated with alternative splicing. Several mechanisms of alternative splicing have been reported, including skipped exons (SE) (a specific exon is excluded from mature mRNA), mutually exclusive exons (MXEs) (choice between two constitutive exons), alternative 3′/5 splicing sites (A3SS/A5SS) (distinct 3′ or 5′ splicing sites are generated in the resulting isoforms), and retained introns (RIs) [42]. RI is the predominant form of alternative splicing in plants and generates transcripts with premature termination codons (PTCs), thus leading to nonsense mRNA decay (NMD) [43]. A total of 1540 differential alternative splicing (DAS) genes were identified, accounting for 4.85% of all DEGs. Overall, the proportion of RIs decreased during cold exposure, while MXEs appeared after 1 d. The proportion of A3SS also significantly increased (Fig. 5a). These results suggest that plants attempt to alter splicing patterns to cope with environmental cues more efficiently.

*MAF1* (*FLM*) and *MAF2* are regulated by alternative splicing. They are homologous to *FLC* and serve as floral repressors during vernalization. However, their expression was upregulated during cold exposure and downregulated following recovery at 22 °C, as expected (Figure S1). Therefore, *MAF1* and *MAF2* are involved in an alternative splicing-mediated thermosensory pathway rather than vernalization. *FLM* regulates flowering through change in the *FLM-β/δ* ratio when plants experience temperature fluctuations between 16 °C and 27 °C [44]. At 4 °C, *FLM-β* was the only upregulated transcript, while *FLM-δ* expression was relatively stable (Fig. 5b), indicating that FLM suppresses flowering in an *FLM-β*-dependent and *FLM-δ*-independent manner under cold but non-freezing conditions. *MAF2* also responds to cold by altering the expression of the sole transcript *MAF2var1* [45].

Regarding specific response pathways, the alternative splicing mechanism significantly affected every known step during cold signaling (Table S5). The cold response starts with signal transduction. The Ca^2+^-permeable cyclic nucleotide-gated channel 5 (*CNGC5*), *CNGC6*, and phytochromes *PHYA* and *PHYB* are the most upstream genes that respond to temperature signals in an alternative splicing-dependent manner (Fig. 5b) [46, 47]. CBL-interacting protein kinase 3 (*CIPK3*) is a crucial kinase for Ca^2+^ signal transduction, and PHYTOCHROME-INTERACTING FACTOR 4 (*PIF4*) and *PIF7* are important factors for thermosensory regulation via phyB; all these factors are regulated through alternative splicing [48–50]. The overall expression levels of *CIPK3*, *PIF4*, and *PIF7* did not change significantly, but their functional transcripts were differentially expressed (Fig. 5b). *BES1*, *EIN4*, and *SPY* are essential regulators of brassinosteroid, ethylene, and GA signaling; *ICE1*, *COR15A*, and *COR27* are the core genes of the cold acclimation pathway; and *ATX1*, *ATX2*, and *FCA* are the chromatin or RNA regulators. All these genes are regulated through alternative splicing (Fig. 5b) [51–56]. Finally, we selected two vernalization genes, namely *VRN5* and *VAL2*, whose overall expression was not upregulated, but the proportion of their sole transcripts was altered (Fig. 5b, S1).

We classified these analyzed genes into three types: change in the proportion of different transcripts, induction of functional transcripts, and synergistic function of all transcripts (Fig. 5b). *CNGC5*, *ICE1*, *EIN4*, *PHYB*, *PIF4*, *RAB8*, *ATX1*, *VRN5*, and *VAL2* belonged to type one, in which the proportions of different transcripts were altered. This type of gene may have antagonistic functions. *FLM*, *MAF2*, *CIPK3*, *COR27*, and *ATX2* belonged to type two, whose transcript expression was distinctly increased; *CNGC6*, *COR15A*, *BES1*, *PHYA*, *PIF7*, and *FCA* belonged to type three, which functioned synergistically. Therefore, alternative splicing is involved in the intricate responses to temperature changes.

## Discussion

### Relationship between cold acclimation and Vernalization

Temperature is an essential environmental stimulus that significantly affects plant growth and development. Plants use various strategies to cope with different durations of cold. Vernalization is a quantitative cold-sensing process that enables plants to flower in warm springs. Simultaneously, plants must also initiate a stress response to cold during vernalization. Cold acclimation enables plants to rapidly adapt to cold temperatures. A time lag of approximately 10 d between the initiation of the two pathways has been proposed [12], and whether these two successive pathways interact under the same cold treatment should be elucidated. The expression of *VIN3* cannot be detected before T14 d. Additionally, a recent study showed that *NTL8*, an upstream regulator of *VIN3*, does not function similarly to other genes involved in cold response, such as *COR47* [36], whose expression pattern was consistent with that of *VIN3* (Fig. 3c). *FLC* was upregulated by cold at T1d. This effect was overridden by vernalization following *VIN3* induction, as *FLC* expression continued to decrease. Core genes of cold stress response *CBF1* and *CBF2* were upregulated at T0.5h (Fig. 3). Thus, the aforementioned time-lag notion was confirmed by *the CBFs* and *VIN3* induction times (Fig. 3). In addition, other genes involved in the cold signaling pathway, such as *CAMTA3*, *MPK3*, *MPK6*, *CIPK7*, *and OST1*, also show increased expression to mediate cold tolerance [57, 58] (Table S5). The independence between cold acclimation and vernalization has been illustrated by the demonstration that core components of the cold-responsive pathway, such as ICE1, HOS1, and IP3-related CVP2, have no effect on *VIN3* expression [59]. The WGCNA results provided complementary verification of the detection of *CBFs*, *ICE1*, *HOS1*, and *CVP2 LIKE 1*(*CVL1*) in modules, while *VIN3* and *FLC* were co-expressed with no genes related to the cold-response pathway.

### Sensors of cold acclimation and Vernalization

Several recent studies have focused on temperature sensors. *COLD1* acts as a cold sensor at 2–4 °C and confers chilling tolerance in Japonica rice [60]. *COLD1* is homologous to *AtGTG1* and *AtGTG2* and functions together with RGA1 to mediate the cold-induced influx of extracellular Ca^2+^ in rice [60]. The expression levels of *GTG1* and *GTG2* decreased within 1 d and returned to normal levels after 14 d (Figure S2). CNGCs are nonspecific cation channels, putatively downstream of *GTG1*, and the expression levels of CNGCs and GTGs were similar (Figure S2) [60]. *PHYB* acts as a thermal sensor at 12–27 °C, sensing temperature change through thermal reversion [61]. At the transcriptional level, *PHYB* senses low temperature via downregulation for 1 d, followed by upregulation under long-term cold conditions (Figure S2). *PIF*s, with known binding sites, are downstream of *PHYB*, and their expression pattern is similar to that of *PHYB* (Figure S2). According to Jung et al. [62], *ELF3* responds to increasing temperatures within 22–27 °C by reversibly forming liquid droplets. Interestingly, the expression pattern of *ELF3* resembled that of *GTG*s or *PHYB*—decreased within 24 h of cold treatment and increased thereafter, and the three reported thermal sensors showed similar expression changes when treated at 4 °C (Figure S2). Therefore, low temperatures may have a similar effect on these thermal sensors at the transcriptional level.

Whether sensors of cold stress and vernalization are the same remains debatable. Suppression of *FLC* expression involves VIN3-dependent and independent pathways [63]. Temperature-dependent growth has been reported as a long-term thermosensor for VIN3-dependent repression of *FLC* [36]. Reduced NTL8 protein accumulation due to slow growth at low temperatures is the major cause of VIN3 accumulation [36]. At the transcriptional level, the expression pattern of *NTL8* resembled that of *VIN3*, being upregulated at 14 d and downregulated at 29 d (Fig. 4f). The effect of cold treatment on *NTL8* mRNA induction in *Col*-FRI background was more obvious than that in the *Col* background [36]. Physical disruption of the FLC-containing gene loop is considered as a sensor of the VIN3-independent pathway of *FLC* repression during the vernalization process [64, 65]. The transcription activity of *FLC* reportedly drops to its lowest level within 1 week. However, *FLC* showed a short-term HOS1-dependent upward trend in response to cold at 1 d (Fig. 3a). This trend can be maintained at 10 d of cold exposure, as recently demonstrated by another transcriptomic analysis [66]. One of the supporting pieces of evidence is that *UPTREAM OF FLC* (*UFC*), which is located 4.7 kb upstream of *FLC*, is suppressed in parallel with *FLC* during vernalization [65]. Indeed, the expression of *UFC* was also upregulated at 1 d and then downregulated thereafter.

### Alternative splicing mediation during cold exposure

Temperature is considered a key factor in the regulation of alternative splicing. Temperature-dependent alternative splicing is associated with various temperature-related processes, such as hibernation in mammals and cold acclimation in plants and fish [67–69]. Calixto et al. [69] found that plants undergo rapid and dynamic alternative splicing in response to short-term cold conditions. With the aim of exploring alternative splicing mediation under long-term cold conditions, we assessed the overall trends of change and regulation of individual genes during 42 d of cold treatment. Alternative splicing was active throughout cold exposure. Many splicing regulation-related genes showed increased expression during cold treatment (Figure S3). Overall, the proportion of the five mechanisms was altered during cold exposure to generate additional functional transcripts, thereby enhancing cold tolerance more efficiently (Fig. 5a). Specific genes whose overall expression did not change may be greatly upregulated as a single transcript. Typically, genes involved in cold acclimation and vernalization respond to cold via three major mechanisms: change in the proportion of different transcripts, induction of functional transcripts, and synergistic function of all transcripts, which enable plants to respond to environmental cues more intricately and efficiently (Fig. 5, S1).

In conclusion, using transcriptomic analysis of the entire vernalization process, we uncovered the independence of cold acclimation and vernalization and further revealed the response networks involved in prolonged cold exposure of plants. Plants tend to alter splicing patterns (decreasing the proportion of RIs) in response to long-term cold, and alternative splicing mechanisms may regulate the entire process of cold response. Thermal sensors exhibit similar expression patterns under non-freezing but cold conditions, and they may sense non-freezing cold at the transcriptional level.

## Materials and methods

### Plant material and growth conditions

*Arabidopsis* FRI-*Col* (*Col-0* with a functional *FRI* allele) was used in this experiment. A functional FRI locus from the Sf2 line was introgressed into Col to construct FRI-Col by the R. Amasino lab [70], and the FRI-Col seeds were provided by Dr. Yuehui He (Shanghai Center for Plant Stress Biology, Chinese Academy of Sciences). Permissions for using these materials were obtained from the Chinese Academy of Sciences. Seeds were surface-sterilized with 75% ethyl alcohol for 1 min, followed by 10% sodium hypochlorite for 15 min, washed six times with sterile water, and stratified at 4 °C for 2 d before being sown on 1/2MS medium. Seedlings were incubated in growth chambers at 22 °C under a 16-8 h day-night period for approximately 2 wk. and then three biological replicates of samples were harvested when they bore four true leaves (T0h). The remaining samples were transferred to an 8-16 h day-night period at 4 °C for 30 min (T0.5h), 1 d (T1d), 14 d (T14d), or 28 d (T28d), and finally harvested. The 29 d samples were subjected to 1 d of recovery at 22 °C (T29d), and the 30 d samples were exposed to cold for 1 d following recovery (T30d). The 42 d samples were subjected to an additional 14 d of treatment at 4 °C under short-day conditions and then harvested (T42d). All the samples were harvested at zeitgeber time 7 (ZT 0 is light-on) except that T0.5h was at ZT 7.5.

### RNA extraction and RNA-seq library construction

Total RNA was extracted using RNAiso Plus (TaKaRa) and processed for mRNA enrichment and rRNA removal. The mRNA was enriched with a polyA tail using magnetic beads with OligodT. Fragmentation buffer was added to break the mRNA into short fragments at high temperature, and the fragmented mRNA was used as the template to synthesize the first-strand cDNA. Next, the second-strand cDNA was synthesized, and the recovered cDNA was purified using a commercial kit. Next, the base “A” was added to the 3 ends of the cDNA, and a linker was connected. The size of the fragment was determined, and the fragments were subjected to PCR amplification. The quality of the constructed library was checked, and the libraries were sequenced.

### DEGs and DAS genes

High-throughput sequencing was performed using the BGISEQ-500 platform. After several data processing steps (including removal of adaptor sequences, null reads, and low-quality reads), pure reads were obtained from the original sequence. After obtaining clean reads, HISAT was used to align the clean reads to the reference genome (GCF_000001735.4_TAIR10.1). Bowtie2 [71] was used to align clean reads to the reference gene sequences, and RSEM [72] was used to calculate the gene expression level of each sample.

rMATS was used to detect DAS genes between different samples and splicing events of the samples. rMATS was used for the analysis of DAS genes based on RNA-seq data. It uses the rMATS statistical model to quantify the expression of alternative splicing events in different samples and then calculates the *P* value with a likelihood-ratio test to indicate whether the two groups of samples are in IncLevel (Inclusion Level), which uses the Benjamini–Hochberg algorithm for correcting the *P* values to obtain the false discovery rate.

### WGCNA

To assess similarities in expression patterns among the groups, we analyzed the transcriptome profiles of biological replicates. The log2-normalized FPKM values of gene expression were input into the WGCNA package in R [73] to generate gene networks. A standard process was used to minimize the noise. An adjacency matrix was constructed using a soft threshold power of 9. Networks were identified using a dynamic tree-cut algorithm with a minimum cluster size of 30 and merging threshold of 0.25. Hub genes were identified based on their eigengene connectivity (KME) [74]. Networks were visualized using Cytoscape v3.5.1 (https://cytoscape.org/).

### qRT-PCR of DEGs

The reliability of DEGs or transcripts identified through RNA-seq was evaluated through qRT-PCR of *FLC*, *VIN3*, *CBF1*, *CBF2*, *MAF1*, and *SOC1*. An Eppendorf Mastercycler Ep RealPlex 2S (Hamburg, Germany) fluorescence quantifier was used. Reactions were performed using the 2× SYBR Green qPCR Master Mix (Bimake), following the manufacturer’s instructions. The specific reaction system contained 10.0 μL of SYBR® Premix Ex Taq™ II, 1.0 μL each of 10 μM forward and reverse primers, 5.0 μL of cDNA template, and 3.0 μL of ddH_2_O. The reaction conditions were as follows: 95 °C for 10 min, followed by 40 cycles of 95 °C for 15 s, 55 °C for 15 s, and 72 °C for 20 s. Finally, gene expression levels were quantified using the 2^-ΔΔCT^ method.

## Supplementary Information

**Additional file 1: Table S1.** DEGs in cluster 1, 2, 3, 4, 5, 6, 7, 8, 9.

**Additional file 2: Table S2.** DEGs in cold stress and vernalization pathways.

**Additional file 3: Table S3.** Information of DEGs in weighted gene co-expression network analysis.

**Additional file 4: Table S4.** DEGs in ERF, LTI and SCC networks.

**Additional file 5: Table S5.** DEGs regulated by alternative splicing mechanism and *CAMTA3*, *MPK3*, *MPK6* and *OST1*.

**Additional file 6: Table S6.** Oligous used as primers in the experiment.

**Additional file 7: Figure S1.** Heatmap showing the expression pattern of selected DEGs. Gene expression data were normalized to Log_2_(FPKM+1); red and blue represent up- and downregulated genes, respectively.

**Additional file 8: Figure S2.** Heatmap showing the expression pattern of selected DEGs. Gene expression data were normalized to Log_2_(FPKM+1); red and blue represent up- and downregulated genes, respectively.

**Additional file 9: Figure S3.** Heatmap showing the expression pattern of selected DEGs. Gene expression data were normalized to Log_2_(FPKM+1); red and blue represent up- and downregulated genes, respectively.

## Data Availability

The datasets generated during the current study were submitted to the NCBI repository, bioproject PRJNA662458. https://www.ncbi.nlm.nih.gov/sra/PRJNA662458.
