# Supplementary figures and images for: Transcriptome analysis reveals Vernalization is independent of cold acclimation in Arabidopsis
Source: BMC Genomics. 2021 Jun 21;22:462. doi: 10.1186/s12864-021-07763-3 (PMC8218483; doi:10.1186/s12864-021-07763-3)

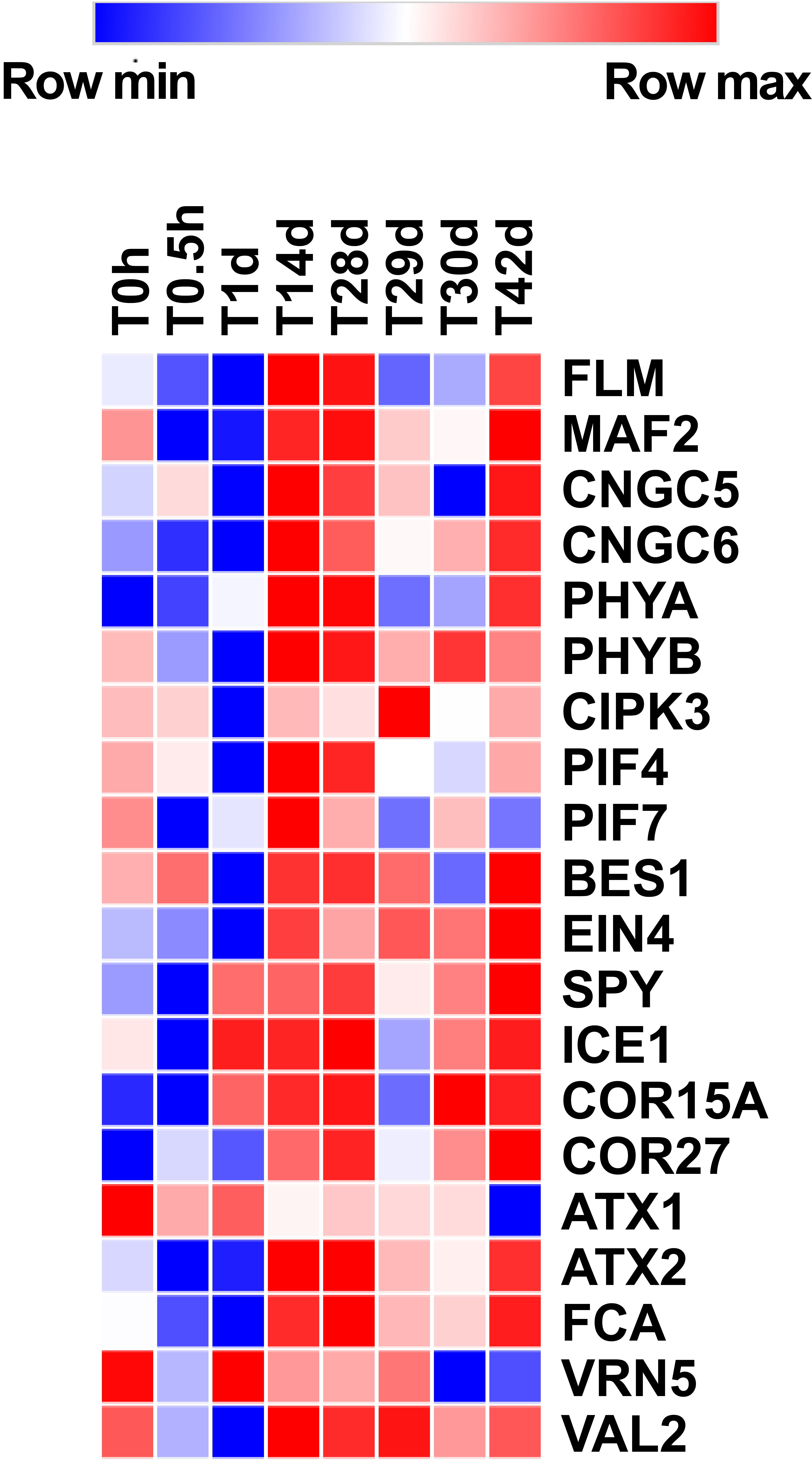

Supplement: Supplementary file 7 — Additional file 7: Figure S1. Heatmap showing the expression pattern of selected DEGs. Gene expression data were normalized to Log2(FPKM+1); red and blue represent up- and downregulated genes, respectively. [file 12864_2021_7763_MOESM7_ESM.png]

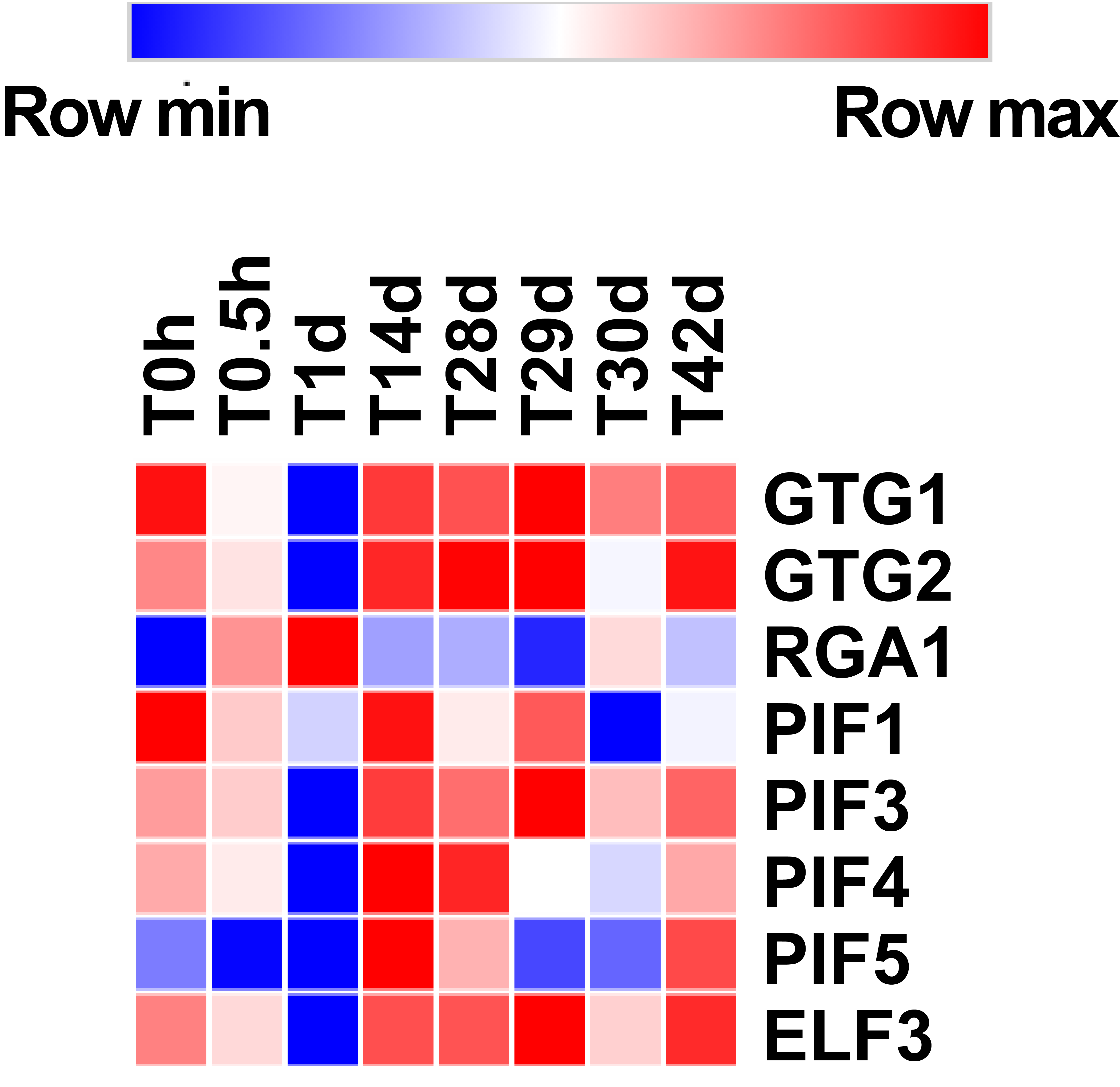

Supplement: Supplementary file 8 — Additional file 8: Figure S2. Heatmap showing the expression pattern of selected DEGs. Gene expression data were normalized to Log2(FPKM+1); red and blue represent up- and downregulated genes, respectively. [file 12864_2021_7763_MOESM8_ESM.png]
